# Case report: A single novel calpain 3 gene variant associated with mild myopathy

**DOI:** 10.3389/fgene.2024.1437859

**Published:** 2024-12-05

**Authors:** Sara Massucco, Paola Fossa, Chiara Fiorillo, Elena Faedo, Chiara Gemelli, Rita Barresi, Michela Ripolone, Serena Patrone, Andrea Gaudio, Paola Mandich, Fabio Gotta, Serena Baratto, Monica Traverso, Livia Pisciotta, Federico Zaottini, Mattia Camera, Elena Scarsi, Marina Grandis

**Affiliations:** ^1^ Department of Neuroscience, Rehabilitation, Ophthalmology, Genetics, Maternal and Child Health (DiNOGMI), University of Genoa, Genova, Italy; ^2^ Department of Pharmacy, Section of Medicinal Chemistry, School of Medical and Pharmaceutical Sciences, University of Genoa, Genova, Italy; ^3^ Paediatric Neurology and Neuromuscular Disorders Unit, University of Genoa and IRCCS Istituto Giannina Gaslini, Genova, Italy; ^4^ IRCCS Ospedale Policlinico San Martino, Genova, Italy; ^5^ IRCCS San Camillo Hospital, Venice, Italy; ^6^ Neuromuscular and Rare Diseases Unit, Department of Neuroscience, Fondazione IRCCS Ca’ Granda Ospedale Maggiore Policlinico, Milan, Italy; ^7^ Center of Translational and Experimental Myology, IRCCS Istituto Giannina Gaslini, Genova, Italy; ^8^ Pediatric Neurology and Muscular Diseases Unit, IRCCS Istituto Giannina Gaslini, Genoa, Italy; ^9^ Department of Internal Medicine (DiMI), School of Medical and Pharmaceutical Sciences, University of Genoa, Genova, Italy

**Keywords:** calpain, calpainopathy, LGMD, LGMDR1, case report

## Abstract

Recessively inherited limb-girdle muscular dystrophy type 1, caused by mutations in the calpain 3 gene, is the most common limb-girdle muscular dystrophy worldwide. Recently, cases of autosomal dominant calpainopathy have been described. A man was referred to our neurological outpatient clinic at the age of 54 for persistent hyperCKemia (>1000 U/l) associated with muscle fatigue and myalgia. Clinical examination revealed mild proximal weakness in the lower limbs. His brother exhibited a moderate increase in serum creatine kinase levels (up to 2000 U/l) without other signs of myopathy. Their father experienced slowly progressive lower limb weakness after the age of 50. The calpain 3 variant c.1478G>A (p.Arg493Gln) in the heterozygous state was identified in both brothers. *In silico* modeling studies predict that this substitution may disrupt protein folding. This represents the first description of the heterozygous p.Arg493Gln calpain 3 variant as a potential cause of mild calpainopathy.

## 1 Introduction

Limb-girdle muscular dystrophy recessive type 1 (LGMDR1, previously LGMD2A), caused by calpain 3 (*CAPN3*) gene variants typically resulting in loss of function of calpain 3 (CAPN3), is the most common recessive LGMD worldwide (Zatz and Starling, 2005; Guglieri et al., 2008; Norwood et al., 2009).

CAPN3 is a skeletal muscle-specific calcium-dependent non-lysosomal cysteine protease identified to interact with the giant sarcomeric protein titin and with filamin C (Richard et al., 1995; Taveau et al., 2003). While the precise role of CAPN3 remains incompletely understood, it appears to encompass various functions, including the assembly and remodeling of contractile proteins in the sarcomere, regulation of calcium efflux from the sarcoplasmic reticulum, facilitation of sarcolemmal repair, and promotion of muscle regeneration (Taveau et al., 2003; Huang et al., 2008; Toral-Ojeda et al., 2016; Hauerslev et al., 2012).

LGMDR1 is characterized by progressive weakness and wasting of proximal muscles, usually involving the pelvic girdle first. Elevated serum creatine kinase (CK) levels and dystrophic changes on muscle biopsy are commonly present. Weakness may be asymmetric, and scapular winging is common, while cardiac involvement is usually absent. Asymptomatic hyperCKemia is also possible, and a wide phenotypic variability exists (Zatz and Starling, 2005).

In recent years, *CAPN3* variants have been identified in families with an autosomal dominant (AD) myopathy, typically characterized by a milder phenotype and later onset (LGMDD4), challenging the exclusive recessive inheritance pattern of this disorder (Vissing et al., 2016; Vissing et al., 2020; Martinez-Thompson et al., 2018; Cerino et al., 2020a; González-Mera et al., 2021; Cerino et al., 2020b).

Vissing et al. reported 37 subjects with a single c.643_663del21 (p.Ser215_Gly221del) in-frame deletion in the *CAPN3* gene, showing an AD transmission across several generations (Vissing et al., 2016). Shortly after, the same heterozygous variant was observed in several additional cases (Martinez-Thompson et al., 2018; Nallamilli et al., 2018). The c.598_612del15 (p.Phe200_Leu204del) in-frame deletion and the novel splice-site c.2440-1G>A (p.Trp814*) mutation have also been associated with LGMDD4 (Cerino et al., 2020b; Mao et al., 2024). Furthermore, cases of late-onset camptocormia associated with the c.759_761del (p.Lys254del) in-frame deletion have been reported (Liewluck and Goodman, 2012; Spinazzi et al., 2021). Cerino et al. identified a single missense variant, c.1333G>A (p.Gly445Arg), in 14 patients with mild myopathy (Cerino et al., 2020a). Additional heterozygous missense *CAPN3* variants were also described: c.700G>A (p.Gly234Arg), c.1327T>C (p.Ser443Pro), c.1333G>A (p.Gly445Arg), c.1661A>C (p.Tyr554Ser), c.1706T>C (p.Phe569Ser), c.1715G>C (p.Arg572Pro), and c.2437G>A (p.Glu813Lys) (Vissing et al., 2020; González-Mera et al., 2021; Şahin et al., 2023).

We herein report a heterozygous missense variant in *CAPN3* associated with hyperCKemia and slowly progressive muscle weakness.

## 2 Case report

A now 69-year-old man was referred to our neuromuscular outpatient clinic at the age of 54 for persistent hyperCKemia [CK > 1000 U/L; most recent CK value: 1175 U/L]. He had been experiencing muscle fatigue and post-exercise myalgia for 3 years. Examination showed mild proximal muscular wasting and weakness, especially in gluteal and hamstring muscles [Medical Research Council score 4/5]. A slight atrophy of the pectoral muscles was also observed, and deep tendon reflexes were weak in all four limbs. The patient exhibited a waddling gait and difficulty climbing stairs, requiring support from the handrail. Gowers’ sign was also present, while no osteoskeletal abnormalities were noted. The echocardiogram, stress electrocardiogram, and cardiology evaluation were all normal.

Following a hyperCKemia protocol (Gemelli et al., 2022), we first ruled out Pompe disease through dried blood spot testing and excluded Myotonic Dystrophy type 2 and copy number variations in the Duchenne muscular dystrophy gene using multiplex ligation-dependent probe amplification (MLPA).

**FIGURE 1:**
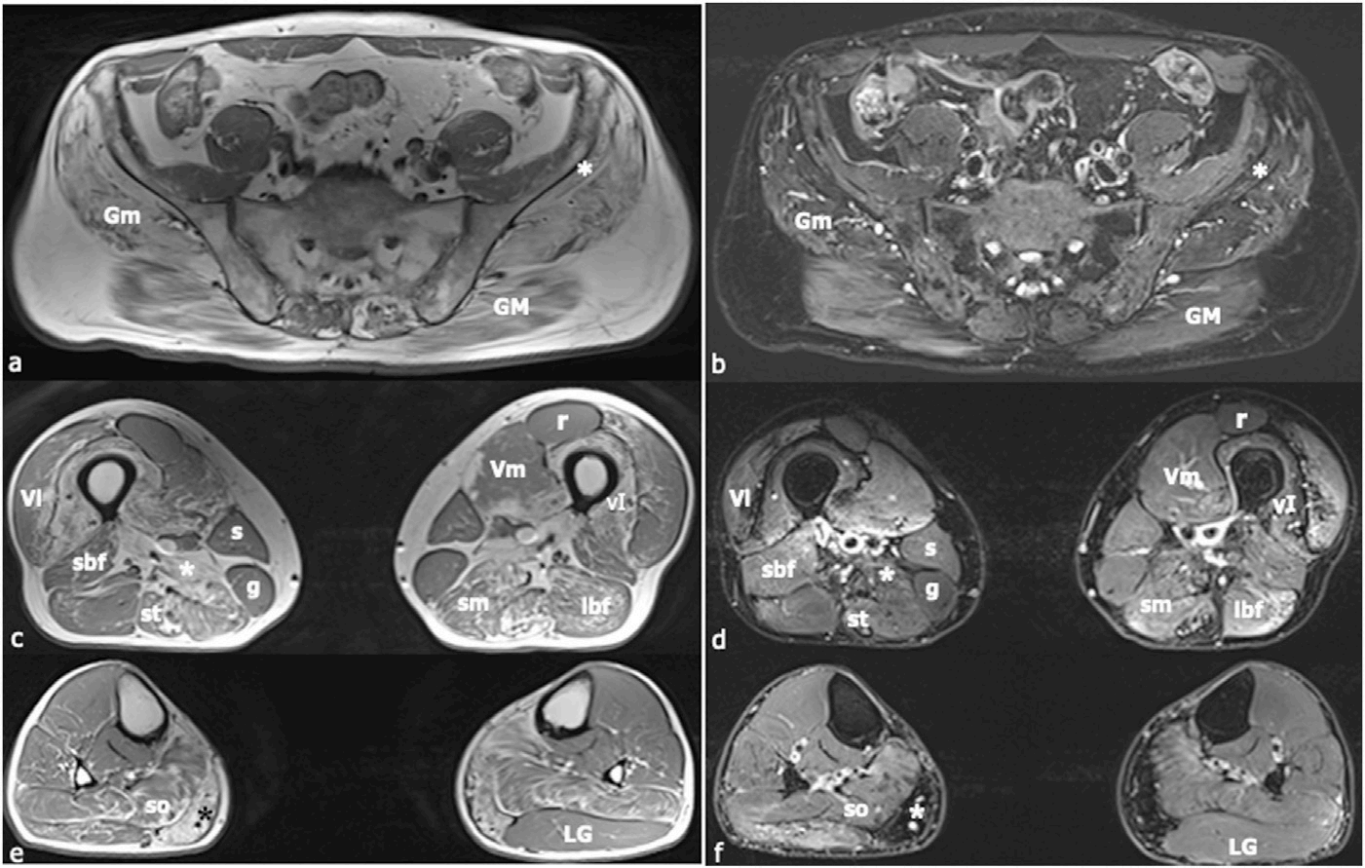
Muscle Magnetic Resonance Imaging (MRI). In **(A)**, **(C)**, and **(E)** axial, T1-weighted images of the lower limbs are shown, whilst in **(B)**, **(D)**, and **(F)** axial T2-weighted images with fat-saturation are displayed. Images **(A)** and **(B)** were acquired at the level of the first sacral vertebra, images **(C)** and **(D)** at the midportion of the thigh, and images **(E)** and **(F)** at the proximal third of the leg. Increased T1-signal related to fibrous and fat infiltration was observed in the gluteus maximus (*GM*), gluteus medius (*Gm*), gluteus minimus [white asterisk in **(A)**, **(B)**], semimebranosus (*sm*), semitendinosus (*st*), adductor magnus [white asterisk in **(C)**, **(D)**], vastus intermedius (*vI*), medial gastrocnemius [black asterisk in **(E)**, white asterisk in **(F)**], and soleus (*so*) muscles. A mild signal intensity increase in the T2-weighted sequence was found in the short head of the biceps femoris (*sbf*), long head of the biceps femoris (*lbf*), semimembranosus (*sm*), and semitendinosus (*st*) muscles, corresponding to muscle necrosis and consequent inflammatory response. The sartorius (*s*), gracilis (*g*), and lateral gastrocnemius (*LG*) muscles did not present any morphological changes. Muscle alteration pattern is consistent with the findings previously described in autosomal dominant calpainopathies. *Vl* = vastus lateralis; *Vm* = vastus medialis; *r* = rectus femoris.

Electromyography findings were normal except for occasional positive potentials in the right gastrocnemius muscle. Lower-limb Magnetic Resonance Imaging (MRI) revealed fatty replacement in proximal muscles, particularly the hip adductors and hamstrings (Figure 1).

**FIGURE 2:**
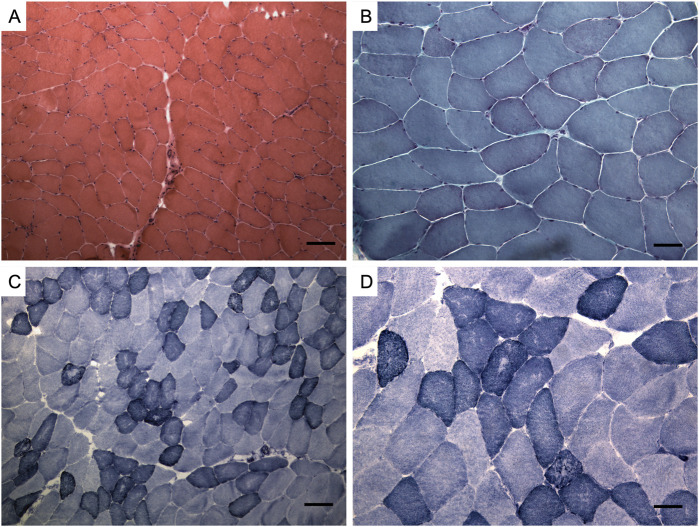
Muscle biopsy. Hematoxylin and eosin staining **(A)**, Modified Gomori trichrome staining **(B)**, Nicotinamide adenine dinucleotide staining (NADH) **(C, D)**. Muscle changes are milder than in limb-girdle muscular dystrophy recessive type 1 (LGMDR1). Observations include a mild fiber caliber variation, rare fibers with internalized nuclei, and fiber splitting. No necrosis or interstitial cellular infiltrates were detected, and vessels and connective tissue appeared normal. Oxidative enzymatic activity was unevenly distributed in some muscle fibers, often displaying moth eaten and core-like features. Bar: **(A, C)** 100 μm, **(B, D)** 50 µm.

A muscle biopsy of the brachial biceps revealed myopathic features with mild fiber size variability and scattered hypotrophic fibers (Figure 2). Immunohistochemistry (caveolin-3, desmin, dystrophin, myotilin, α-sarcoglycan, γ-sarcoglycan) showed normal staining patterns (not shown).

The patient’s younger brother was examined at the age of 59 due to a moderate, persistent increase in serum CK levels (up to 2000 U/L). The neurological examination was unremarkable; specifically, muscle tone, trophism, and strength were preserved, deep tendon reflexes were normal, and gait showed no pathological findings. Nonetheless, CK levels remained persistently elevated, with a most recent value of 345 U/L.

Their father experienced slowly worsening lower-limb weakness from age 50. Notably, he experienced significant difficulty climbing stairs. Neurological examination revealed atrophy and weakness in both quadriceps with Gowers’ sign, waddling gait, and absent deep tendon reflexes in the lower limbs. Serum CK levels ranged from 377 to 484 U/L. Electromyography showed fibrous resistance during needle insertion in the right quadriceps and left deltoid, along with submaximal interference at maximum effort and reduced potential duration. In the left rectus femoris, fibrous resistance was also noted upon needle insertion, with occasional fibrillation potentials, small positive potentials, and a slight increase in irregular potentials. Muscle MRI showed signs of fatty infiltration in the gluteal and thigh muscles bilaterally, with a normal appearance of the medial portion of the vastus medialis and the sartorius and gracilis muscles. At age 52, he underwent a quadriceps muscle biopsy, which showed findings suggestive of muscular dystrophy. Specifically, moderate variation in fiber caliber was observed, with numerous hypertrophic fibers adjacent to atrophic ones, many fibers with centralized nuclei, and some ring fibers. There was also a slight increase in connective tissue between individual fibers. Cardiological evaluations remained persistently unremarkable.

Next-Generation Sequencing (NGS) of a panel of 43 genes associated with hyperCKemia revealed the *CAPN3* missense variant c.1478G>A (p.Arg493Gln) in the heterozygous state in both siblings. This variant is classified as likely pathogenic according to the American College of Medical Genetics and Genomics guidelines. MLPA did not detect any copy number changes in the *CAPN3* gene. To rule out the possibility that variants in other genes could explain the clinical picture, whole-exome sequencing was performed on both siblings. It did not reveal any additional candidate genes explaining the myopathy. We could not assess the segregation of the variant in the father due to his death from prostate adenocarcinoma at the age of 88.

Western blot analysis of the proband’s muscle protein lysate using anti-CALP12A2 and anti-CALP2C4 antibodies revealed normal CAPN3 expression (not shown).

### 2.1 *In silico* modeling studies

**FIGURE 3:**
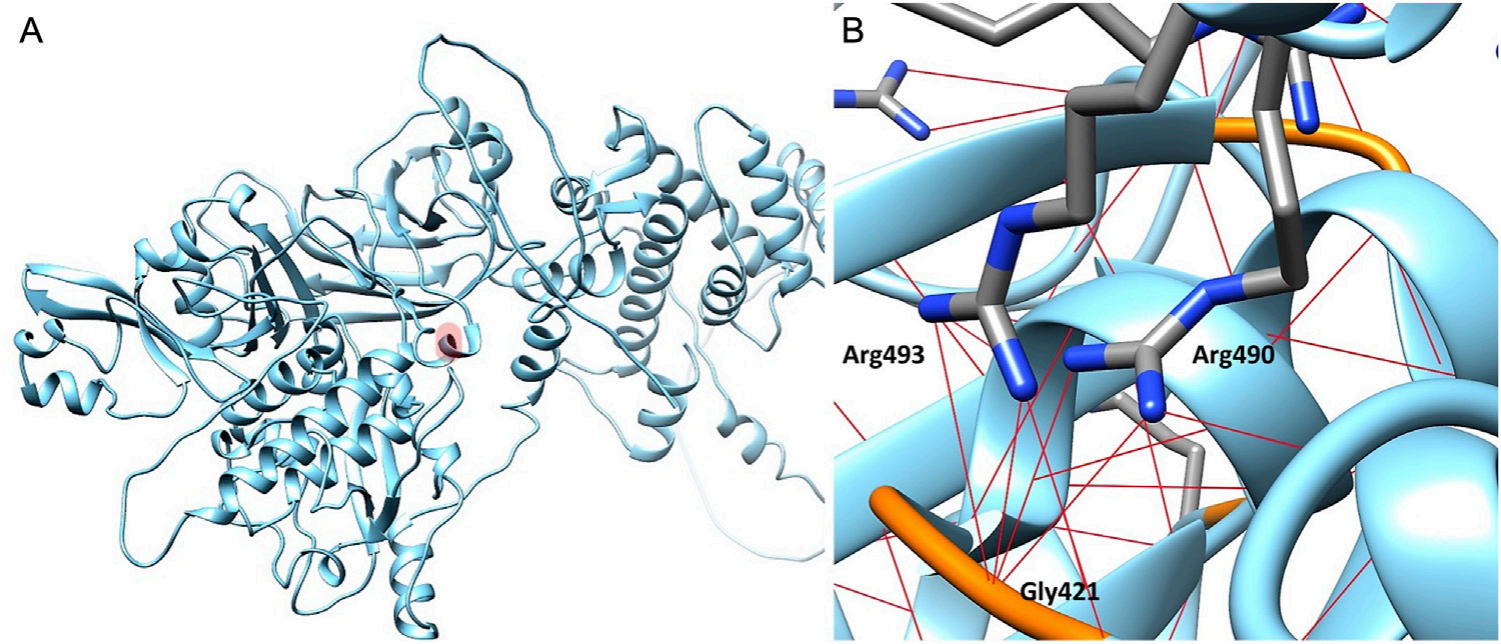
Calpain-3 3D model. **(A)** Residue Arg493 is highlighted in light red. **(B)** Focus on the Arg493 subregion in Calpain-3 (ribbon). Arginine residues are represented as capped sticks, Gly421 residue is depicted in orange, and hydrogen bonds are shown as red dashed lines.

The impact of the Arg493Gln mutation on CAPN3 was investigated by building a CAPN3D model using Alphafold (Jumper et al., 2021), based on the structure of CAPN2 available in the Protein Data Bank (PDB) (PDB code: 3BOW) (Berman et al., 2000), along with five CAPN3 structures (PDB codes: 4OHK, 6BDT, 6BPG, 6BJD, 6BKJ). All experimental structures were determined by X-rays and are of good quality, with resolutions ranging from 2.45 to 3.20 Å. The model allowed comparison between wild-type and mutated proteins, focusing on the area surrounding residue Arg493. In native CAPN3, Arg493, located near Arg490, forms three hydrogen (H) bonds with the Gly221 backbone, similar to Arg490. As almost all Arg residues in CAPN3, the basic side chain of Arg493, according to the model, plays a critical role in establishing tighter interactions within specific protein sub-regions to ensure proper protein folding. The mutation to Gln, lacking the basic guanidinium moiety on its side chain, negatively affects the extensive H-bond network in the region surrounding residue 493. Gln is unable to participate in all the H-bond interactions facilitated by Arg (Figure 3). Therefore, according to the 3D model, the mutation adversely affects protein folding.

## 3 Discussion

The novel heterozygous p.Arg493Gln variant detected in our patients, impacting a highly conserved residue, is predicted to affect protein folding according to our *in silico* studies. *In silico* modeling is a useful tool for investigating in-depth biological, biochemical, and genetic mechanisms. Several *in silico* studies have supported its role in predicting the functional effects of gene variants (Duarte et al., 2018). Notably, previous research on CAPN3 variants has benefited from computational approaches (Vissing et al., 2020; González-Mera et al., 2021; Banerjee et al., 2024; Aguti et al., 2024).

Support for the pathogenicity of our variant comes from family transmission, the mild late-onset phenotype, muscle MRI findings, *in silico* modeling, and the absence of other pathogenic variants in the siblings.

Unfortunately, we were unable to confirm the segregation of the p.Arg493Gln variant in the father of our patients, which certainly represents a limitation of the study. Since he presented a clinical picture compatible with LGMD, the transmission within our family is potentially AD. However, we cannot demonstrate this with certainty due to the lack of more extensive segregation studies. Furthermore, although whole-exome sequencing did not identify variants in other genes potentially associated with myopathy, we cannot definitively exclude the presence of intronic variants since the entire genome was not sequenced.

Western blot analysis showed normal CAPN3 expression in our patient’s muscle, contradicting the typical loss-of-function pattern in LGMDD4. For the c.643_663del21 variant, Western blotting revealed normal expression of mutated messenger ribonucleic acid (mRNA) and loss of CAPN3 muscular expression, suggesting a dominant negative effect with a loss-of-function mechanism (Vissing et al., 2016). The single missense mutation c.1715G>C (p.Arg572Pro) also results in the loss of the autolytic and proteolytic properties of CAPN3 (Vissing et al., 2020). Both the p.Gly445Arg mutation and our variant are located within the calpain beta-sandwich domain of the protein, which is known to interact with the penta-EF-hand domain and is involved in CAPN3 dimerization (Cerino et al., 2020a). Regarding the literature data, Western blot analysis showed normal levels of CAPN3 expression in two out of four patients with the p.Gly445Arg mutation, but the variant impaired CAPN3 autolytic activity and intra/intermolecular autolysis in immortalized human myoblasts (Cerino et al., 2020a). Another nearby *CAPN3* variant, c.1343G>A (p.Arg448His), retains catalytic activity but disrupts titin interaction (Ermolova et al., 2011). In our patients, *in silico* modeling studies have shown that substituting Arginine with Glutamine at position 493 prevents the formation of the H-bonds typically formed by Arginine, disrupting protein folding.

In LGMDR1, MRI scans reveal typical involvement of the adductor magnus and posterior thigh muscles, with relatively spared anterior thigh muscles (Barp et al., 2020; Mercuri et al., 2005). Similar patterns of muscle replacement by fat, particularly in the hamstring muscles like the semitendinosus and semimembranosus muscles, have been noted in AD calpainopathies (Vissing et al., 2016; Cerino et al., 2020a), and are consistent with the findings in our case.

The nonspecific myopathic changes observed in muscle histology are also consistent with AD calpainopathy. In the case series of LGMDD4 reported by Vissing and colleagues, dystrophic changes on muscle biopsy did not reach the extent observed in LGMDR1, with milder nonspecific myopathic features like an increased number of internalized nuclei and mild to moderate variation in fiber size being reported (Vissing et al., 2016).

The younger brother presented hyperCKemia in the absence of other signs or symptoms of myopathy. Despite its lack of specificity, hyperCKemia is a common finding in LGMDD4. Moreover, rare cases of exertional rhabdomyolysis possibly related to LGMDD4 have been described (Cerino et al., 2020a).

In the case of other neuromuscular disorders, such as Charcot-Marie-Tooth disease caused by mutations in the ganglioside-induced differentiation-associated protein-1 gene, both AD and autosomal recessive patterns of transmission have been identified, with AD forms typically associated with milder phenotypes (Pezzini et al., 2016). As regards recessive myopathies, heterozygous variants in the ryanodine receptor isoform-1, α‐sarcoglycan, and dystroglycan genes can also result in a milder phenotype (Traverso et al., 2024).

The increasing use of NGS techniques, even in patients with isolated or paucisymptomatic hyperCKemia, could result in the detection of heterozygous variants in *CAPN3*. In cases with mild phenotypes, after ruling out duplications/deletions on the other allele and confirming that the variant affects the protein structure or function, LGMDD4 may be considered.

## 4 Conclusion

This is the first description of the heterozygous p.Arg493Gln variant in CAPN3 as a potential cause of mild calpainopathy. According to *in silico* modeling studies, substituting arginine with glutamine at position 493 is predicted to interfere with protein folding. Remarkably, whole-exome sequencing only revealed the c.1478G>A (p.Arg493Gln) mutation in *CAPN3* as a potential explanation for the myopathy of our patients. Furthermore, the combination of clinical presentation and findings from muscle MRI, muscle biopsy, and *in silico* modeling studies strongly supports calpainopathy as the underlying cause.

## Data Availability

The datasets presented in this article are not readily available because of ethical and privacy restrictions. Requests to access the datasets should be directed to the corresponding author.

## Abbreviations

AD, autosomal dominant; CAPN3, calpain 3; CK, creatine kinase; LGMD, limb-girdle muscular dystrophy; LGMDR1, limb-girdle muscular dystrophy recessive type 1; LGMDD4, limb-girdle muscular dystrophy dominant type 4; MLPA, multiplex ligation-dependent probe amplification; MRI, Magnetic Resonance Imaging; mRNA, messenger ribonucleic acid; NGS, Next-Generation Sequencing.
